# CXCR2: A Novel Mediator of Mammary Tumor Bone Metastasis

**DOI:** 10.3390/ijms20051237

**Published:** 2019-03-12

**Authors:** Bhawna Sharma, Kalyan C. Nannuru, Sugandha Saxena, Michelle L. Varney, Rakesh K. Singh

**Affiliations:** 1 Gilead Biosciences, Foster City, CA 94404, USA; bhanu.micro@gmail.com; 2 Regeneron Pharmaceuticals Inc., Terrytown, NY 10591, USA; knannuru@gmail.com; 3 Department of Pathology and Microbiology, University of Nebraska Medical Center, Omaha, NE 68198-5845, USA; sugandha.saxena@unmc.edu; 4 Department of Internal Medicine, University of Nebraska Medical Center, Omaha, NE 68198-5845, USA; mvarney@unmc.edu

**Keywords:** CXCR2, metastasis, bone microenvironment

## Abstract

Most breast cancer patients die due to bone metastasis. Although metastasis accounts for 5% of the breast cancer cases, it is responsible for most of the deaths. Sometimes even before the detection of a primary tumor, most of the patients have bone and lymph node metastasis. Moreover, at the time of death, breast cancer patients have the bulk of the tumor burden in their bones. Therapy options are available for the treatment of primary tumors, but there are minimal options for treating breast cancer patients who have bone metastasis. C-X-C motif chemokine receptor type 2 (CXCR2) receptor-mediated signaling has been shown to play a critical role during bone-related inflammations and its ligands C-X-C motif chemokine ligand 6 (CXCL6) and 8 (CXCL8) aid in the resorption of bone during bone metastasis. In this study, we tested the hypothesis that CXCR2 contributes to mammary tumor-induced osteolysis and bone metastasis. In the present study, we examined the role of both tumor cell-derived and host-derived CXCR2 in influencing mammary tumor cell bone metastasis. For understanding the role of tumor cell-derived CXCR2, we utilized Cl66 CXCR2 knockdown (Cl66-shCXCR2) and Cl66-Control cells (Cl66-Control) and observed a significant decrease in tumor growth and tumor-induced osteolysis in Cl66-shCXCR2 cells in comparison with the Cl66-Control cells. Next, for understanding the role of host-derived CXCR2, we utilized mice with genomic knockdown of CXCR2 (Cxcr2^−/−^) and injected Cl66-Luciferase (Cl66-Luc) or 4T1-Luciferase (4T1-Luc) cells. We observed decreased bone destruction and metastasis in the bone of Cxcr2^−/−^ mice. Our data suggest the importance of both tumor cell- and host-derived CXCR2 signaling in the bone metastasis of breast cancer cells.

## 1. Introduction

Breast cancer is the most frequently diagnosed cancer in women in the United States with an estimated 266,120 new cases in 2018, which represents more than one-quarter (30%) of the total estimated new cancer cases [1]. The five-year relative survival for women suffering from localized breast cancer is 99%, but women diagnosed with metastatic breast cancer disease have a five-year relative survival of only 27% [2]. Thus, the primary tumor itself is not the leading cause of death, but rather a metastasis to distant organs and lack of response to conventional chemotherapeutic treatments. Currently, therapeutic options are available to eradicate the primary tumor, but there are only a few recognized therapies for distant breast cancer cases.

Breast cancer is known to metastasize to lymph nodes, lung, liver, and bone. Breast cancer cells show a strong predilection for metastases to bone. Tumor-induced osteolysis is a predominant feature in breast cancer bone metastasis [3] and also causes skeletal lesions including pathological fracture, intractable bone pain, nerve compression and hypercalcemia [3]. These complications not only decrease the quality of life but also in some cases lead to mortality.

The arrival of tumor cells at the bone microenvironment initiates a vicious cycle of bi-directional interaction between tumor cells and stromal cells of bone microenvironment. Tumor cells produce various factors to stimulate bone matrix resorption, which in turn releases factors that favor the growth of tumor cells. Evidence in the literature suggests that chemokines and receptors influence various aspects of cancer development [4,5,6,7]. Chemokines not only have been shown to support tumor growth but also have been implicated in their progression and the establishment of tumor cells at distant organ sites [8]. C-X-C motif chemokine receptor type 2 (CXCR2) is a chemokine receptor known to regulate inflammatory responses during infections and wound healing. Chemokines, which bind to CXCR2, are CXC chemokines with the ELR+ motif, namely, CXCL1, 2, 3, 5, 6, 7 and 8. These chemokines have chemoattractant properties for leukocytes and lymphocytes to mediate their migration toward inflammatory sites and secondary lymphoid organs. Some of them have also been shown to promote angiogenesis [8,9], proliferation [10,11,12,13] and aids chemotherapy resistance in various cancer types [8,9,13,14]. CXCL8, a ligand for CXCR2, has been shown to regulate osteoclast activation both in a RANKL dependent and independent pathway during breast cancer bone metastasis [15,16]. A recent study suggests that during autoantibody-mediated arthritis, CXCR2 has a critical role in recruiting neutrophils and this helps in the development of the disease [17]. CXCR2 binds to all these chemokines, which have been shown to promote metastasis in various cancer types, and this appeared to us as a promising molecule to study to assess bone metastasis in breast cancer.

In this study, we investigated the role of both tumor and host CXCR2 expression in tumor-induced osteolysis using syngenic mouse models. We observed a significant reduction in tumor-induced osteolysis and osteoclast homing at the tumor-bone interface in mice injected with Cl66 CXCR2 knockdown (Cl66-shCXCR2) cells in comparison with mice injected with Cl66-Control cells (Cl66-Control). Also, we observed a similar reduction in tumor growth and osteolysis in CXCR2 knockout mice injected with murine breast cancer cells than in the wild type Control mice. These results suggest that both tumor-derived CXCR2 and host CXCR2 may play a critical role in tumor-induced osteolysis.

## 2. Results

### 2.1. Tumor Cell CXCR2 Signaling Promotes Tumor Growth in the Bone Microenvironment

**Figure 1:**
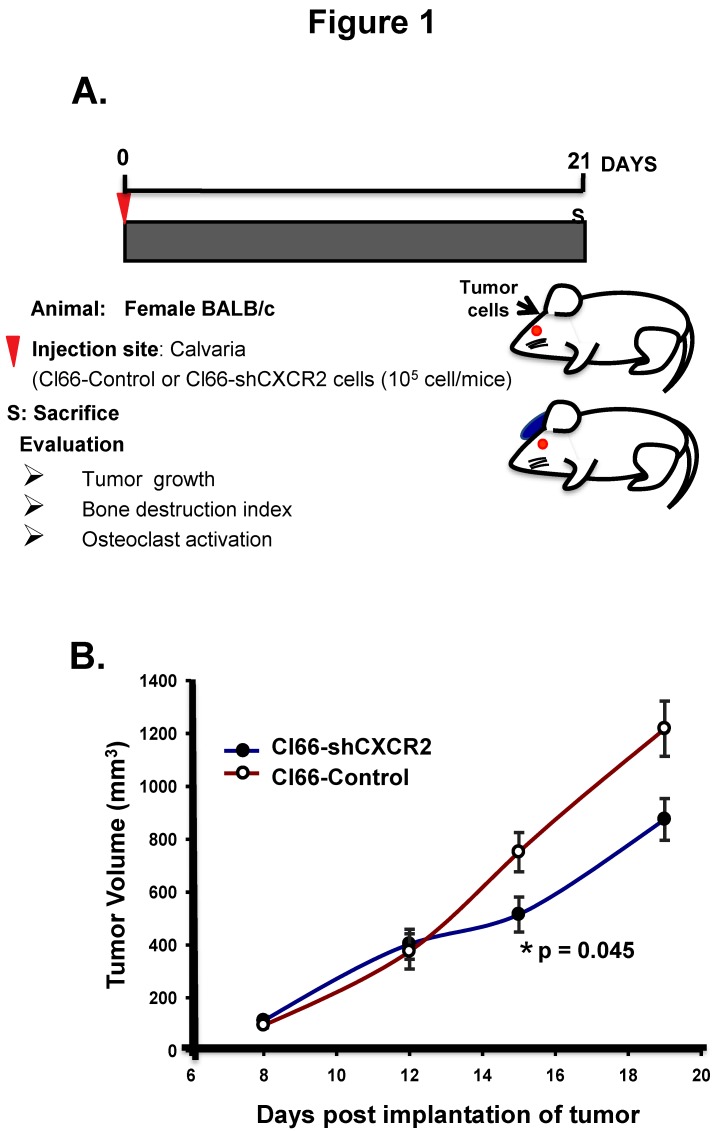
Downregulation of CXCR2 in tumor cells reduces calvarial tumor growth. (**A**) Schematic representation of the tumor cell injections in Balb/c mice. Injection of Cl66-Control or Cl66-shCXCR2 cells mixed and suspended in 25 μL of Hank’s Balanced Salt solution and 25 μL of growth factor reduced matrigel on the dorsal side on calvaria of Balb/c mice using a 23 gauge needle is marked as day 1. Mice were monitored for 21 days for tumor growth and sacrificed. (**B**) The graph shows the growth kinetics of tumor formed by Cl66-Control and Cl66-shCXCR2 cells on the calvaria of Balb/c mice. Statistical analysis was performed using the Mann-Whitney Rank Sum Test with * *p* = 0.045 and *n* = 5 per group.

To determine the role of CXCR2 signaling in the tumor-bone interface, we utilized an experimental osteolytic bone metastasis model; implanting the breast cancer cells on the dorsal calvaria of mice and evaluating the tumor growth, bone destruction index, and osteoclast activation (Figure 1A). With this aim, we implanted breast cancer Cl66-Control and -CXCR2 knockdown cells on the dorsal calvaria of mice and first evaluated the tumor growth. We observed a significant decrease in tumor growth in mice implanted with Cl66-shCXCR2 cells (*p* = 0.045) in comparison with Cl66-Control cells (Figure 1B).

### 2.2. Knockdown of CXCR2 in Tumor Cells Diminishes Bone Destruction in Mice

**Figure 2:**
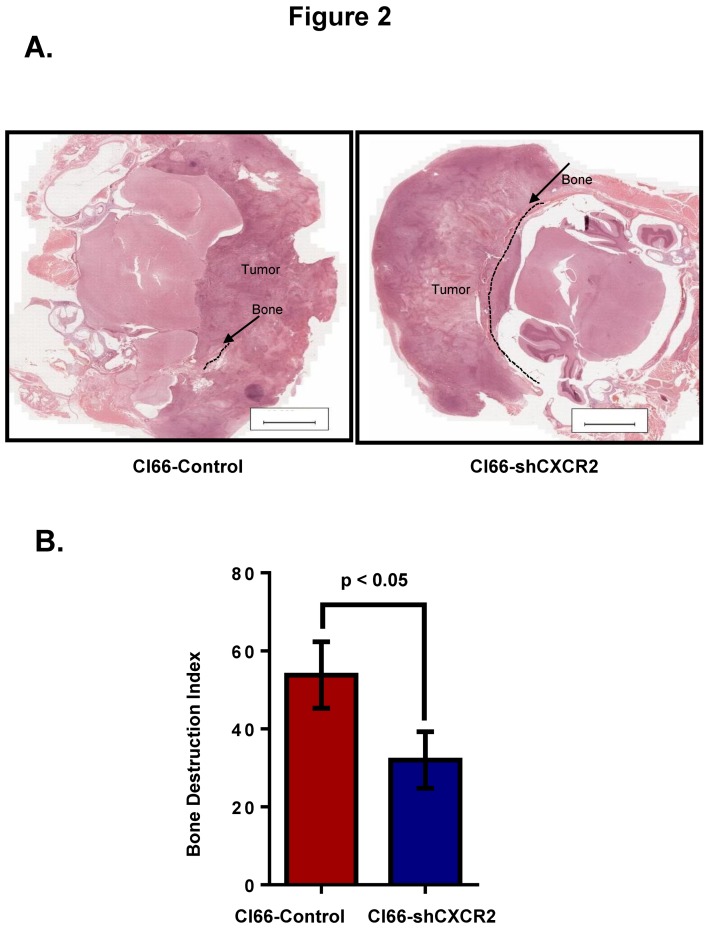
CXCR2 downregulation in Cl66 cells significantly decreased tumor-induced osteolysis. (**A**) Representative images show H&E staining demonstrating intact cranial bone in Cl66-shCXCR2 group in comparison with severe bone destruction in Cl66-Control group. Scale bar represents 10,000 μm. (**B**) Bone destruction index was used to measure the severity of the lesions. Bar graph showing significantly lower bone destruction index in Cl66-shCXCR2 group (32 ± 5) in comparison with Cl66-Control group (54 ± 6) (*n* = 5, *p* < 0.05).

Second, we wanted to evaluate how the tumor CXCR2 affects bone damage during breast cancer bone metastasis. Towards this aim, we collected tumor bone sections from mice after three weeks of breast cancer cell implantation. We observed reduced osteolysis at the tumor-bone interface in a group of mice injected with Cl66-shCXCR2 in comparison with the group of mice injected with the Cl66-Control cells (Figure 2A). We also determined the severity of these lesions (tumor-induced osteolysis) by calculating the bone destruction index and observed significant inhibition (*p* < 0.05) of tumor-induced osteolysis in mice implanted with Cl66-shCXCR2 in comparison with mice implanted with Cl66-Control cells (Figure 2B).

### 2.3. Tumor CXCR2 Signaling Enhances Osteoclast Activation during Bone Metastasis

**Figure 3:**
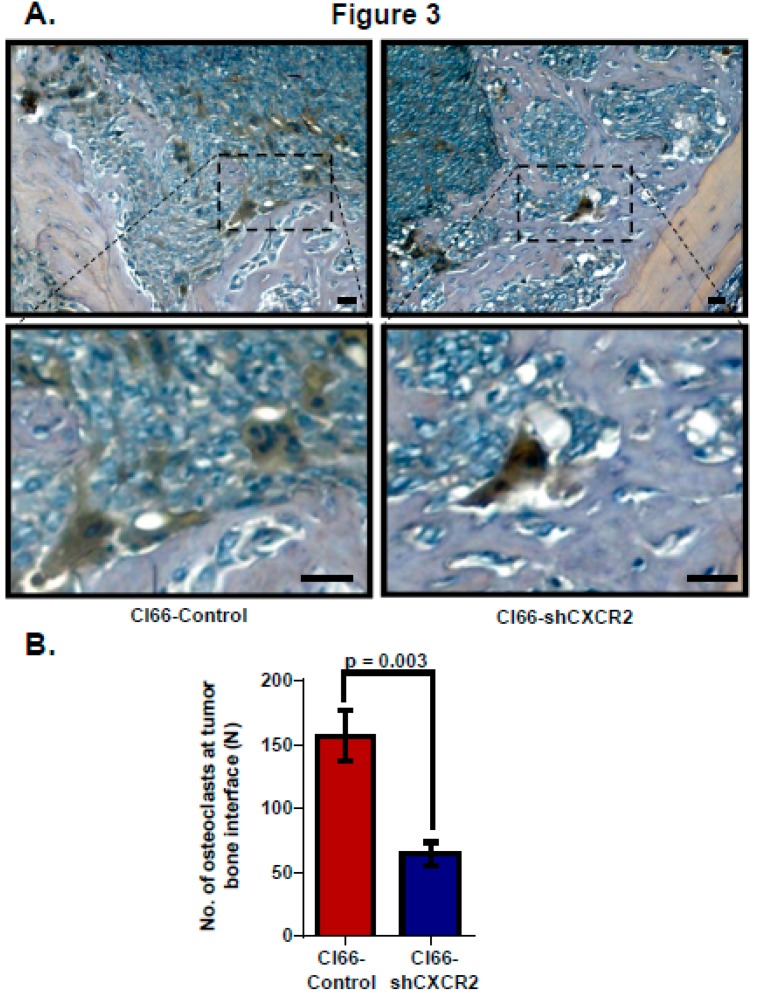
CXCR2 downregulation in tumor cells lowers activated osteoclast number at the tumor-bone interface. (**A**) Representative images of osteolytic activity as determined by TRAP staining at the tumor-bone interface from Cl66-Control and Cl66-shCXCR2 tumor-bearing mice. Scale bar represents 10 μm (**B**) Bar graph showing a significantly lower number of TRAP-positive osteoclasts in Cl66-shCXCR2 (65 ± 9) in comparison with the Cl66-Control tumor group (157 ± 12) (*n* = 5, *p* = 0.003) at the tumor-bone interface.

Lastly, to evaluate tumor CXCR2 signaling, we analyzed osteoclast homing at the tumor-bone interface in the sections of mice injected with Cl66-Control or Cl66-shCXCR2 cells. We analyzed osteoclasts at the tumor-bone interface using Tartrate-resistant acid phosphatase (TRAP) staining, which stains explicitly multinucleated alkaline phosphatase producing activated osteoclasts. Like the difference we observed in tumor-induced osteolysis, TRAP-positive multinucleated osteoclasts homing was lower in Cl66-shCXCR2 implanted mice in comparison with Cl66-Control implanted mice (Figure 3A). We observed 65 ± 9 osteoclasts homed at the TB interface in the CL66-shCXCR2 tumor group compared to 157 ± 19 osteoclasts in the Control tumor group (Figure 3B).

### 2.4. Host CXCR2 Mediates Tumor Cell Growth in the Bone Microenvironment

**Figure 4:**
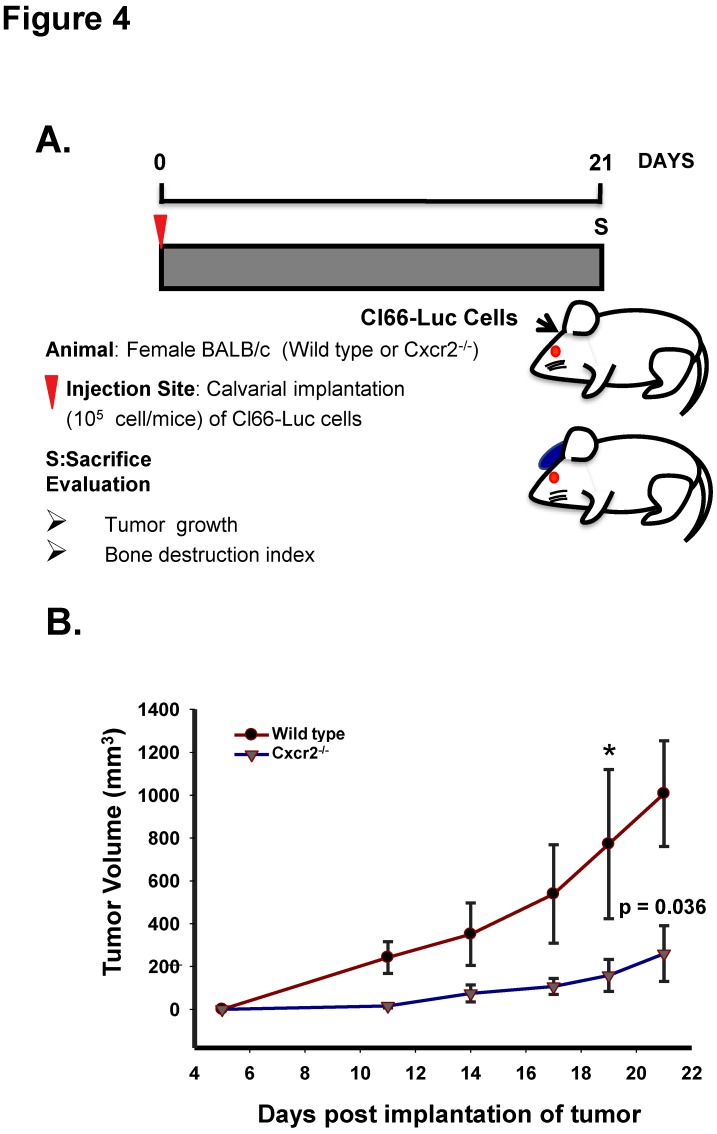
Host CXCR2 promotes tumor cell growth in the bone microenvironment. (**A**) Schematic diagram of the Cl66-Luc cell injection in calvaria of wild type and Cxcr2^−/−^ mice. (**B**) The graph shows the kinetics of tumor volume formed by Cl66-Luc cells in the bone microenvironment of the wild type and CXCR2 ^−/−^ mice. Statistical analysis was done using the Mann-Whitney Rank Sum Test with * *p* = 0.036 and *n* = 5/group.

CXCR2 has been shown to be expressed during inflammation of bone-related diseases [17,18] and is present on the surface of mesenchymal cells [19]. As mesenchymal cells lead to the formation of various cells of the bone microenvironment, we wanted to evaluate how host CXCR2 affects mammary tumor cells growth in the bone microenvironment. With this aim, we implanted Cl66-Luc cells on the dorsal side of calvaria of WT, and Cxcr2^−/−^ mice (Figure 4A) and observed a significant reduction in the growth kinetics of tumor in Cxcr2^−/−^ mice in comparison with the wild type mice (Figure 4B). Our result suggests that host CXCR2 positively influences tumor cell growth in the bone microenvironment.

### 2.5. Host mCxcr2 Knockdown Decreases Bone Destruction

**Figure 5:**
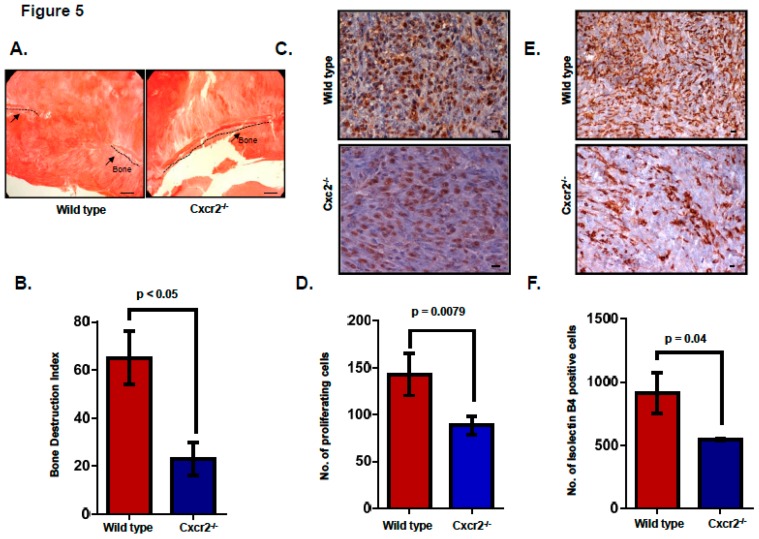
Host Cxcr2 deficiency reduced tumor-induced osteolysis. (**A**) Representative images show H&E staining demonstrating the intact cranial bone in Cxcr2^−/−^ mice in comparison with severe bone destruction in wild type mice. Scale bar represents 100 μm (**B**) Bar graph shows significantly lower bone destruction index in Cxcr2^−/−^ mice in comparison with wild type mice (*n* = 5, *p* < 0.05). (**C**) Representative images show immunohistochemical staining for PCNA cells demonstrating higher cell proliferating cells in the tumor of the wild type mice in comparison with the Cxcr2^−/−^ mice. Scale bar represents 10 μm. (**D**) Bar graph demonstrates a lower number of proliferating cells in Cxcr2^−/−^ mice in comparison with wild type mice (*n* = 5, *p* = 0.0079). (**E**) Representative images show immunohistochemical staining for isolectin B4 positive cells demonstrating higher microvessel density in the tumor of the wild type mice in comparison with the Cxcr2^−/−^ mice. Scale bar represents 10 μm. (**F**) Bar graph demonstrates lower microvessel density at the tumor-bone interface in Cxcr2^−/−^ mice in comparison with wild type mice. (*n* = 5, *p* = 0.04).

Recent literature suggests that neutrophils express CXCR2 [20,21] and are the critical mediators of metastasis present in the bone microenvironment. CXCL8, one of the chemokines that bind to the CXCR2 receptor has been shown to recruit neutrophils which then leads to the generation of soluble RANKL and hence osteoclast activation [22]. Sundaram et al. report that CXCL5, another CXCR2 ligand, can mediate RANKL expression which is known for its role in osteoclast activation during bone metastasis of breast cancer cells [23]. Based on knowledge accumulated from the above literature, we wanted to examine the importance of host CXCR2 in accentuating bone destruction during tumor progression. As described for tumor-derived CXCR2, we determined the severity of osteolysis by calculating the bone destruction index and observed significant inhibition (*p* < 0.05) of osteolysis in Cxcr2^−/−^ mice implanted with Cl66-Luc cells in comparison with the wild type mice (Figure 5A,B) suggesting that the scarcity of CXCR2 dependent signaling in the host may have abrogated the activation of osteoclasts.

Next, as CXCR2 is a well-known player in cancer cell survival and tumor angiogenesis, we wanted to examine how loss of host Cxcr2 influences the in situ cell proliferation and microvessel density in the tumor-bone interface. Towards this goal, we performed immunohistochemical analysis using PCNA, a cell proliferation marker, and isolectin B4, marker for microvessel density, in wild type and Cxcr2^−/−^ mice tumors. We observed a significantly higher frequency of proliferating cells (Figure 5C,D, *p* = 0.0079) and microvessel density (Figure 5E,F, *p* = 0.04) in tumors growing in wild type mice in comparison with Cxcr2^−/−^ mice.

### 2.6. Host Cxcr2 Influences Mammary Tumor Cell Bone Metastasis

**Figure 6:**
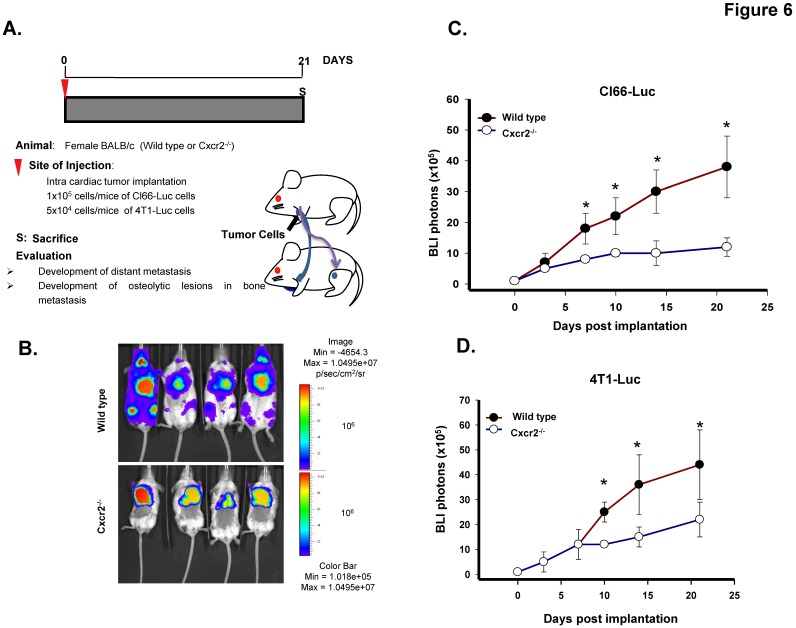
Host Cxcr2 influences mammary tumor bone metastasis. (**A**) Diagram depicts the schematic representation of intracardiac tumor cell injection in wild type and Cxcr2^−/−^ mice. (**B**) In vivo luminescent images of the wild type and Cxcr2^−/−^ group showing the higher spread of Cl66-Luc cells in the whole body of the wild type mice in comparison with the Cxcr2^−/−^ mice. (**C**) Quantification of bone metastasis burden from Cl66-Luc cells injected intracardially in wild type and Cxcr2^−/−^ mice based on whole body BLI imaging. The wild type mice group showed a higher tumor burden than the Cxcr2^−/−^ mice group. The * sign indicates p value < 0.05. (**D**) Quantification of bone metastasis burden from 4T1-Luc cells injected intracardially in wild type and Cxcr2^−/−^ mice based on whole body BLI imaging in wild type and Cxcr2^−/−^ mice. The wild type mice group showed a higher metastatic tumor burden than Cxcr2^−/−^ mice group. The * sign indicates *p* value < 0.05.

Lastly, to determine whether host Cxcr2 status can influence the mammary tumor cell bone metastasis, we injected Cl66-Luc cells intracardially in the wild type and Cxcr2^−/−^ mice and monitored the spread of tumor cells in real time (Figure 6A). We observed reduced luciferase expression in hind limbs and the forelimbs of Cxcr2^−/−^ mice than those of wild type mice, suggesting that host Cxcr2 plays a crucial role in directing tumor cells toward bones (Figure 6B,C). Moreover, to further confirm our findings we injected 4T-Luc cells intracardially in the wild type and Cxcr2^−/−^ mice. We observed similar findings with the 4T1-Luc as with the Cl66-Luc cells (Figure 6D) suggesting that once tumor cells are in the bloodstream, it is the Cxcr2 status of the host which determines their localization in the body. With the 4T1-Luc tumor cell model, in the Cxcr2^−/−^ group, except for one mouse we did not observe any bone metastasis in the rest of the mice, whereas, in the wild type group, 4 out of 5 mice developed bone metastasis.

## 3. Discussion

This study evaluates the role of CXCR2 in breast cancer bone metastasis by analyzing both tumor-derived and host-derived CXCR2 in the bone microenvironment. Recent literature demonstrates the role of CXCR2 ligands in cancer and metastasis, such as CXCL6, one of the CXCR2 ligands that contributes to osteoclast differentiation and activation in a RANKL-dependent pathway [24]. Another ligand, CXCL8, which binds to CXCR2 has been shown to influence bone metastasis in a RANKL-dependent and independent manner in breast cancer [15,16]. A recent study also reports the contribution of CXCR2 ligands CXCL1 and CXCL2 to osteolysis in metastatic prostate cancer [25]. Although the current literature suggests the importance of CXCR2 ligands in bone metastasis, there is a gap in our knowledge about the significance of the CXCR2 receptor itself concerning bone metastasis of breast cancer.

In the present study, we examined the role of both tumor-derived and host-derived CXCR2 in breast cancer bone metastasis. To evaluate the importance of tumor-derived CXCR2 in tumor-induced osteolysis, we implanted Cl66-shCXCR2 and Cl66-Control cells on the calvaria of mice. We preferred the implantation of breast cancer cells on calvaria bone than other available bone metastasis model because previously our group demonstrated that implantation of breast cancer cell lines on the calvaria of syngeneic animals induced osteolytic changes in the bone microenvironment [26,27]. We observed that knockdown of CXCR2 in Cl66 mammary tumor cells decreased the ability of tumor cell to grow on calvaria bone and hence resulted in reduced tumor burden. We also observed reduced tumor-induced osteolysis and osteoclast activation in the presence of CXCR2 knockdown Cl66 tumor cells in comparison with the Cl66-Control cells. Our previous report demonstrates that implantation of Cl66-shCXCR2 and Cl66-Contol tumor cells in the mammary fat pad of female BALB/c mice did not result in any significant difference in the tumor volume between the groups [9]. Thus, our results suggest that the tumor cells require CXCR2 expression for their ability to survive in the bone microenvironment and the effect of depletion of CXCR2 on tumor growth is dependent on the host microenvironment.

To determine the role of host-derived CXCR2, we used mice with a genetically modified CXCR2 background. Similar to our finding in tumor-derived CXCR2, we observed a significant decrease in tumor growth and osteolysis in tumors formed by Cl66-Luc cells injected in Cxcr2^−/−^ mice in comparison with Cl66-Luc cells injected in the wild type mice. Earlier we demonstrated that orthotopic injection of Cl66 and 4T1 cell lines in the mammary fat pad of wild type and Cxcr2^−/−^ mice resulted in decreased tumor growth in Cxcr2^−/−^ mice in comparison with the Control mice [12]. We observed an increase in the secretion of CXCR2 ligands in Cxcr2^−/−^ tumor-bearing mice in comparison with the wild type tumor-bearing mice [12].

The non-functional condition of CXCR2 is known to result in lack of neutrophil recruitment or activation in inflammatory diseases [28], which can also be a possible explanation of suppressed tumor growth of Cl66 cells in Cxcr2^−/−^ mice in comparison with the wild type mice. We have reported the similar observation of decreased tumor-associated granulocytes, tumor-associated macrophages and myeloid-derived suppressor cells in tumors generated from orthotopic injection of Cl66 cells in the mammary fat pad Cxcr2^−/−^ mice in comparison with the wild type mice [12]. Also, the immune component in Cxcr2^−/−^ mice does not display defects in the functionality [29]; therefore, the deficiency is only in the recruitment of immune cells to the tumor site and not systemic.

Lastly, we injected Cl66-Luc cells intracardially in the wild type and Cxcr2^−/−^ mice and observed the lower seeding of tumor cells in the bones of Cxcr2^−/−^ in comparison with the wild type, thereby suggesting that the expression of CXCR2 in the host determines its inhabitation by tumor cells and establishment of bone metastasis. To determine the reproducibility and rule out cell line-specific observations with the Cl66 tumor cell model, we used luciferase-expressing 4T1-Luc cells another syngenic and more aggressive cell lines than Cl66 [30]. We observed significantly lower bone metastasis in the Cxcr2^−/−^ mice in comparison with the wild type mice. Both the cell types used in the study express CXCR2 ligands [9].

The bone microenvironment is composed of various types of cells, which include endothelial cells, fibroblast, and immune cells. The bone marrow mesenchymal stem cells give rise to stromal cells, which eventually differentiate to form various cell types, namely, fibroblast, adipocytes, osteoblasts. Several factors influence tumor-stromal interaction during bone metastasis including metalloproteinase, cathepsins, growth factor, chemokines, and chemokine receptors [31]. These factors not only help the migration of tumor cells to the bone but also support their growth in the bone microenvironment [32]. All the mesenchymal, stromal and most of the transient cells have also been shown to express CXCR2 receptor both in healthy and diseased conditions [20,33]. Fibroblasts in the stroma of the bone secrete matrix metallopeptidase-2 (MMP2) in an inactive state and have been shown to promote bone metastasis in the presence of cancer cells, which activates MMP2 [34]. Cancer cells stimulate osteoclasts in a RANKL-dependent manner and hence aid in bone resorption [35]. Similarly, endothelial cells help in providing a niche for the tumor cells in the bone microenvironment [36]. Apart from stromal cells, transient cells like T-cells also promote bone metastasis [37]. Most of the reports in breast cancer bone metastasis evaluating chemokines CXCL6 and CXCL8 focus on CXCR1, expressed by osteoclasts [16]. However, there is minimal literature investigating the role of CXCR2 in bone metastasis of breast cancer. A CXCR2 ligand, CXCL8, has been shown to be an important player in osteoclast activation and bone metastasis in breast cancer [15,38]. The present report demonstrates the role of CXCR2 in the process of breast cancer metastasizing to the bone. However, our current investigation lacks the mechanistic insight into how host cell types or molecules in the bone interact with CXCR2 during this process.

Previous reports from our laboratory suggest the role of tumor-derived CXCR2 in mammary tumor growth and lung metastasis [9,11]. We also reported that targeting CXCR2 makes tumor cells sensitive towards chemotherapy, again suggesting the importance of CXCR2 signaling in controlling mammary tumor growth and metastasis. This study is an extension of the previous studies and implicates that CXCR2 signaling is also crucial for bone metastasis of mammary tumor cells. Taken together, these studies provide direct evidence of tumor and host-derived CXCR2 in breast cancer progression and metastasis. Other scientific groups also report that CXCR2 expression by the tumor cells promotes tumor cell invasion [32] and metastasis formation [39]. Taken together, our studies implicate targeting CXCR2 as a novel approach for breast cancer treatment. CXCR2 blocking with small molecular antagonists or receptor antagonists could have therapeutic importance in reducing the metastatic disease progression in breast cancer. Moreover, a recent clinical trial in patients with severe asthma and sputum neutrophils has found that the CXCR2 antagonist SCH527123 is safe to use in clinical settings [40]. However, CXCR2 can affect different signaling pathways [10,41], and more investigations are needed to define the major signaling pathways affected upon CXCR2 depletion in mammary tumor-induced bone destruction, growth and metastasis.

## 4. Materials and Methods

### 4.1. Cell Culture

We utilized the murine mammary adenocarcinoma cell lines CI66 and 4T1 (kind gift from Dr. Fred Miller, Karmanos Cancer Institute, Detroit, OH). The details of generation of Cl66-Control and Cl66-shCXCR2 are described elsewhere [9]. We maintained Cl66-Control, Cl66-shCXCR2, Cl66-Luc, and 4T1-Luciferase (4T1-Luc) cells in Dulbecco’s Modified Eagle Media (DMEM) (Mediatech, Hendon, VA, USA) with 5% fetal bovine serum (Sigma, St. Louis, MO, USA), 1% vitamins, 1% l-glutamine and 0.08% gentamycin (Invitrogen, Carlsbad, CA, USA). We supplemented Cl66-Luc and 4T1-Luc cells with blasticidin (15 μg/mL) as a selection marker. Cell lines were authenticated and tested for mycoplasma contamination using a kit, MycoAlert (Cambrex Bio Science Rockland. Inc, Rockland, ME, USA).

### 4.2. Tumor Cell Implantation and Bone Metastasis

We purchased female BALB/c mice (6–8 weeks old) from the National Cancer Institute, and Cxcr2^−/−^ mice from Jackson laboratories. We maintained the mice under specific pathogen-free conditions. The Institutional Animal Care and Use Committee, in the University of Nebraska Medical Center, approved all the procedures and we performed the procedures according to the institutional guidelines. Mice were kept anesthetized during the whole procedure using isoflurane. Cl66-Control or Cl66-shCXCR2 cells (10^5^ cells mixed with growth factor reduced Matrigel) were implanted on the dorsal skin flap over the calvaria of 6–8 week old female Balb/c mice to study tumor growth and bone destruction index concerning tumor-derived CXCR2. We implanted Cl66-Luc cells (10^5^ cells/mice) on calvaria of the 8-week old female Balb/c mice having either wild type or Cxcr2^−/−^ for the assessment of host CXCR2 role in promoting bone deterioration. The details of the generation of whole body Cxcr2^−/−^ mice are described elsewhere [11]. We injected Cl66-Luc in the left cardiac ventricle of wild type or Cxcr2^−/−^ mice to access the homing of mammary tumor cells toward bones. We measured tumor growth twice a week for calvaria implantation. Tumor volume was calculated using the formula π/6 × (smaller diameter)^2^ × (larger diameter). At the end of the study, tumors resected from mice were fixed in formalin, embedded in paraffin and processed for histopathological evaluation and immunohistochemistry.

Bone metastasis was monitored in mice using an experimental bone metastasis model. 4T1-Luc cells (5 × 10^4^) or Cl66-Luc (1 × 10^5^) cells constitutively expressing firefly luciferase were suspended in 50 μL of sterile PBS and injected into the left cardiac ventricle of 6–8 weeks old female Balb/C mice under isoflurane anesthesia. We evaluated disease progression and dissemination to the bone by noninvasive bioluminescence imaging (BLI) using the IVIS Imaging System (Xenogen, Los Angeles, CA, USA) and measured total photon flux (photons/sec) from fixed regions of interest (ROI) over the entire mouse.

### 4.3. Bone Destruction Index

Twenty-one days post-implantation mice were sacrificed and examined for osteolytic lesions. To calculate the bone destruction index, we stained the sections of the tumor-bone region with hematoxylin and eosin [42]. Briefly, the length of the destroyed bone was calculated and was divided by the total length of the tumor-bone interface. The obtained ratio was then multiplied by 100 to obtain the bone destruction index.

### 4.4. Immunohistochemical Analysis

TRAP staining was performed to detect activated osteoclasts in vivo according to the manufacturer’s instructions (Sigma Chemicals, St. Louis, MO, USA). We examined the immunostained sections for quantitative analysis, under a Nikon light microscope, and assessed the number of positive cells at a magnification of 400× for each lesion.

Immunohistochemical analysis was performed to determine in situ cell proliferation and microvessel density as previously described [9]. In brief, 6-μm thick tumor sections were deparaffinized by xylenes and ethanol and blocked for 30 min. Tumor sections were incubated overnight in a humid chamber with an anti-PCNA antibody or biotinylated mouse anti-GS-IB4 (isolectin from *Griffonia simplicifolia*; 1:50; Vector Laboratories, Burlingame, CA, USA) antibody. Immunoreactivity was detected using the ABC Elite kit and DAB substrate (Vector Laboratories, Burlingame, CA, USA) as per the manufacturer’s instructions. A reddish brown precipitate indicated a positive reaction. Negative controls had all reagents except antibody. The number of microvessels was quantitated microscopically with a 5 × 5 reticle grid (Klarmann Rulings, Litchfield, NH, USA) using 400× objective (250 µm total area).

### 4.5. Statistical Analysis

In vivo analysis was performed using the Mann-Whitney *U*-test and paired t-test using Sigma Plot 11. All the values are expressed as mean ± SEM. A *p*-value ≤ 0.05 was considered statistically significant.
